# Impaired Lymphatic Drainage and Interstitial Inflammatory Stasis in Chronic Musculoskeletal and Idiopathic Pain Syndromes: Exploring a Novel Mechanism

**DOI:** 10.3389/fpain.2021.691740

**Published:** 2021-08-23

**Authors:** Brian Tuckey, John Srbely, Grant Rigney, Meena Vythilingam, Jay Shah

**Affiliations:** ^1^Department of Physical Therapy, Tuckey and Associates Physical Therapy, Frederick, MD, United States; ^2^Department of Human Health and Nutritional Sciences, University of Guelph, ON, Canada; ^3^Department of Psychiatry, Oxford University, Oxford, United Kingdom; ^4^Department of Health and Human Services, Center for Health Innovation, Office of the Assistant Secretary for Health, Washington, DC, United States; ^5^Department of Rehabilitation Medicine, Clinical Center, National Institutes of Health, Bethesda, MD, United States

**Keywords:** interstitial inflammatory stasis, cytokines, lymphatic dysfunction, counterstrain techniques, myofascial pain and dysfunction, idiopathic diseases, fascia

## Abstract

A normal functioning lymphatic pump mechanism and unimpaired venous drainage are required for the body to remove inflammatory mediators from the extracellular compartment. Impaired vascular perfusion and/or lymphatic drainage may result in the accumulation of inflammatory substances in the interstitium, creating continuous nociceptor activation and related pathophysiological states including central sensitization and neuroinflammation. We hypothesize that following trauma and/or immune responses, inflammatory mediators may become entrapped in the recently discovered interstitial, pre-lymphatic pathways and/or initial lymphatic vessels. The ensuing interstitial inflammatory stasis is a pathophysiological state, created by specific pro-inflammatory cytokine secretion including tumor necrosis factor alpha, interleukin 6, and interleukin 1b. These cytokines can disable the local lymphatic pump mechanism, impair vascular perfusion via sympathetic activation and, following transforming growth factor beta 1 expression, may lead to additional stasis through direct fascial compression of pre-lymphatic pathways. These mechanisms, when combined with other known pathophysiological processes, enable us to describe a persistent feed-forward loop capable of creating and maintaining chronic pain syndromes. The potential for concomitant visceral and/or vascular dysfunction, initiated and maintained by the same feed-forward inflammatory mechanism, is also described.

## Introduction

Chronic pain is the leading cause of disability with up to 49% of the population experiencing pain <3 months duration. The estimated cost of chronic pain and associated opioid use disorder in the USA is currently between $560 and 635 billion annually (1). Chronic pain is positively correlated with age (2) and, given the rapidly aging demographic, the burden of chronic pain will continue to impose significant challenges to our healthcare system.

Myofascial pain syndrome (MPS) is among the most common, yet least understood forms of chronic musculoskeletal pain, and is a frequent cause of primary care physician and pain clinic visitation (1, 2). Few people live without experiencing muscle pain following injury, overuse, strain, or trauma. Although pain associated with MPS frequently resolves in a few weeks, in some cases it can persist long after the inciting event and/or spread to distant, uninjured tissues (3, 4). Although MPS is typically characterized by the expression of pain localized to myofascial tissues, it is also associated with a broad and growing profile of *non-musculoskeletal* symptoms including fatigue, sleep disturbance, and visceral pain syndromes (5). These associations suggest a shared pathophysiology between MPS and several common idiopathic conditions (e.g., visceral pain syndromes). The pathophysiological mechanisms underlying this association, however, are not fully understood and remain largely undescribed.

It is well-established that persistent, peripheral nociceptive sources can initiate, maintain, and perpetuate chronic pain states. This occurs, in part, through central mechanisms including retrograde inflammation produced by dorsal root reflexes (6), and/or areas of secondary hyperalgesia produced by glial cell neuroinflammation (3). However, in idiopathic *peripherally generated* chronic pain, our understanding of the pathological processes that generate and maintain ongoing nociceptive input is limited. Examples include whiplash associated disorders which present with pain, proprioceptive and autonomic-linked symptoms despite a lack of correlative pathological evidence on computer tomography and/or magnetic resonance imaging [for review see (7–10)]. Additionally, existing pain hypotheses are limited in their ability to address many of the pathophysiological findings common to both chronic pain and idiopathic visceral/vascular syndromes. This includes elevated levels of plasma and interstitial pro-inflammatory cytokines in myofascial (11, 12) and visceral pain syndromes (13), and evidence of sympathetic nerve activation (SNA) in MPS (14–16), visceral disease (17, 18), and vascular disorders (19). Microvascular disturbances and impaired lymphatic function have also been identified in both MPS (20) and visceral disease (21), supporting the concept of a shared pathophysiology.

Considering the limitations in current understanding, we hypothesize that elevated pro-inflammatory cytokine levels, through specific pathophysiological mechanisms, adversely impact vascular hemodynamics and lymphatic function in the extracellular compartment. Impaired venous and lymphatic drainage can create a state of *inflammatory interstitial stasis* (IIS), which results in ongoing nociceptive bombardment of the dorsal horn (central sensitization). Recent anatomical discovery and advances in pre-clinical and clinical research, enable us to further elucidate the potential pathophysiological factors involved in this process. This includes contraction of fascial myofibroblasts following local TGF-b1 expression (22) which we hypothesize can cause pre-lymphatic/lymphatic vessel contraction and/or fibrosis. And the effect of specific pro-inflammatory cytokines including tumor necrosis factor alpha (TNF-a), interleukin-6 (IL-6) and interleukin (IL-1b) in cessation of the normal lymphatic pump mechanism (23), the development of chronic pain states (24) and the creation of long-term microvascular disturbance following stimulation of segmentally linked somato/visceral-sympathetic reflexes (23, 24).

**Figure 1:**
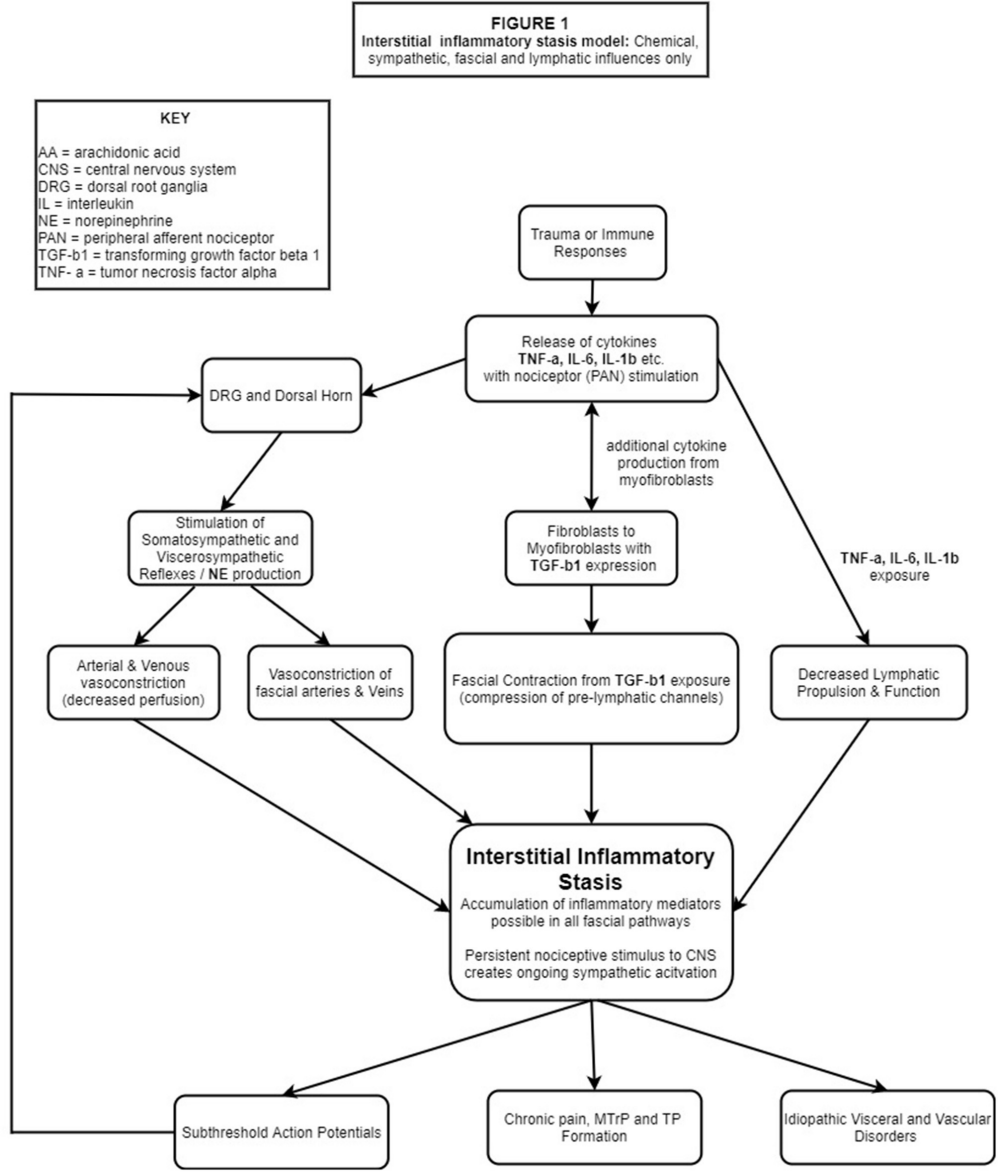
Trauma and/ or immune responses lead to PC production, most notably IL-1β, IL-6 and TNF-α. PANs of multiple tissues embedded in the ECM are stimulated, transporting these substances to the DRG and DH where glial cells are stimulated leading to central and peripheral neuroinflammation/sensitization. Nociceptive bombardment stimulates somato/visceral-sympathetic reflexes causing the release of NE, resulting in peripheral vasoconstriction (including fascial vasculature) while the cytokines IL-1β, IL-6, TNF-α which deactivate the local lymphatic pump mechanism and simultaneously stimulate fibroblasts to differentiate into myofibroblasts. TGF-b1 released by fibroblasts & myofibroblasts, causes contraction of fascial tissues compressing pre-lymphatic pathways. Impaired hemodynamics from vasoconstriction, deactivation of the lymphatic pump mechanism and compression of pre-lymphatic pathways create areas of hypoxia and IIS. Continued PAN stimulation results in a pathophysiological feed-forward loop of lymphatic stasis, nociceptor stimulation and sympathetic activation which manifests in chronic pain, sub-threshold action potentials and idiopathic visceral/vascular dysfunction.

**Figure 2:**
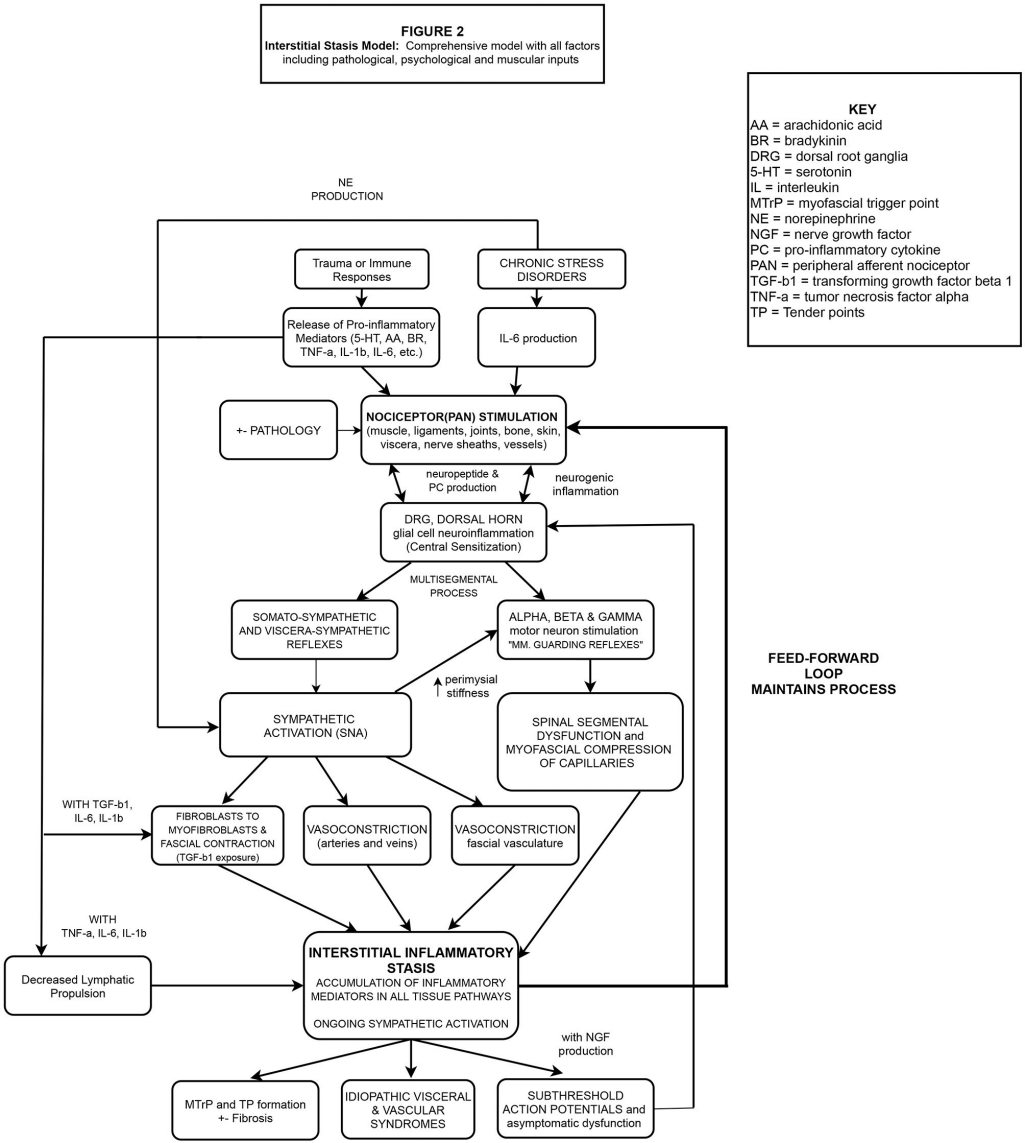
Nociceptive bombardment (various sources) produces PCs including IL-1β, IL-6, TNF-α etc. PANs of multiple tissues embedded in the ECM are stimulated, transporting these substances to the DRG, causing antidromic release of neuropeptides from the DRG into the injured and neurosegmentally linked tissues, exacerbating the response beyond the region of primary hyperalgesia. Glial cells in the DH are stimulated leading to central and peripheral neuroinflammation/sensitization. PAN entry into the DH at multiple levels alters the activity of alpha and gamma motor neurons, creating multi-segmental muscle guarding reflexes, and myofascial compression of pre-lymphatic pathways. Simultaneously, somato/visceral-sympathetic reflexes are stimulated, causing the release of NE, resulting in peripheral vasoconstriction (including fascial vasculature) while cytokines IL-1β, IL-6, TNF-α deactivate local lymphatic propulsion and stimulate fibroblasts to differentiate into myofibroblasts. TGF-b1 released by fibroblasts & myofibroblasts, creates local fascial contraction, perimysial stiffness (gamma motor activation) and compression of pre-lymphatic pathways. Due to the combined mechanisms, areas of hypoxia and inflammatory stasis develop which continuously stimulate local PANs. A pathophysiological feed-forward loop of lymphatic stasis, nociceptor stimulation and SNA manifests in chronic pain, sub-threshold action potentials and idiopathic visceral/vascular dysfunction.

These concepts, including others critical to our IIS hypothesis, will be described in the sections to follow and presented schematically in flowchart format. Figure 1 (flowchart 1) specifically highlights the fascial, sympathetic, and lymphatic pathophysiological mechanisms related to IIS. Figure 2 is a *comprehensive* flowchart which incorporates the concepts in Figure 1 and additional, previously documented mechanisms, that may contribute to the development of IIS.

### Pro-Inflammatory Mediators and Peripheral Afferent Nociceptors

Tissue injury and/or inflammation leads to the local release of algogenic substances including glutamate, serotonin, bradykinin, adenosine triphosphate, protons (low pH), Substance P, nerve growth factor (NGF), and norepinephrine (NE) all of which are transmitted to the central nervous system by primary afferent nociceptors (nociceptors) [for review see Willard (25)]. Nociceptors have unmyelinated free nerve endings that terminate peripherally in the extracellular matrix (ECM), and respond to both inflammatory and mechanical stimuli (26, 27). Virtually all tissues are innervated by nociceptors including fascia (28), tendons (29), blood vessels (30, 31), nerve sheaths (32), ligaments, menisci, synovium, bone (33), visceral tissues or capsules in the case of solid organs (34), vertebral discz (35) and meninges (36). Primary afferent nociceptors enter the dorsal root segmentally, where they trifurcate forming ascending, descending and segmental level fibers. Thus, these small caliber fibers can influence the segmental level of entry and several segments above and below (37). This anatomical structure enables singular activated nociceptors to have heterosegmental nociceptive and reflexive impact. Research specifically highlights the role of visceral afferents in pain production as their activity is synaptically transmitted deep in the dorsal horn to convergent viscero-somatic neurons, which receive nociceptive input from the skin and deep somatic tissues of the corresponding dermatomes, myotomes and sclerotomes (38). Additionally, injury and/or immune responses will result in the production of pro-inflammatory cytokines from various cells, including endothelial cells, macrophages, dendritic cells, and fibroblasts. These substances lower nociceptor activation thresholds in the periphery (25) and, if persistent, can create structural and/or functional changes in the spinal cord including central sensitization (39). Thus, clinical consideration must be given to viral, infectious, traumatic, post-surgical and/or overuse histories as each can facilitate the cellular release of pro-inflammatory cytokines that result in nociceptor activation.

### Neurogenic Inflammation

Persistent nociceptive bombardment of the dorsal horn leads to primary afferent depolarization of convergent somatosensory pathways (40) and dorsal root reflexes which result in neurogenic inflammation or the retrograde release of proinflammatory neuropeptides including substance P and calcitonin gene-related peptide CGRP, into peripheral tissues (6). In support of this concept, IL-1β injections into the dorsal root ganglia (DRG) and dorsal horn are able to induce secondary hyperalgesia, via retrograde inflammation, in the intraperitoneal, intracerebroventricular and intra-plantar tissues of rats (41, 42). This central to peripheral mechanism is a separate phenomenon from spinal glial cell neuroinflammation and expands the inflammatory process into contiguous, non-injured peripheral tissues, creating regions of secondary hyperalgesia (pain experienced outside the original injury site) (3). Glial cell neuroinflammation occurs from nociceptive signals derived from muscle (43), joint (44) and/or visceral (45) tissues and can initiate the transition from acute to chronic pain states following central sensitization (46).

Pro-inflammatory cytokines generated by trauma or immune responses can also be transported *from the periphery, via* axonal or non-axonal mechanisms, to the DRG and dorsal horn, facilitating the induction of central sensitization (47), which has important implications to the concept of IIS. Additionally, Xie 2006, demonstrated that, once inflamed, the DRG not only produces pro-inflammatory cytokines but also decreases its production of anti-inflammatory cytokines (48), which further exacerbates the peripheral inflammatory process. Importantly, studies indicate that specific pro-inflammatory cytokines including IL-1β, IL-6, and TNF-α, are particularly associated with glial cell neuroinflammation and chronic pain states (49).

### The Role of Muscle Guarding Reflexes in the Pathophysiology of Trigger Points

Muscle guarding reflexes are the body's protective, involuntary motor responses to reduce nociception (50). Stimulation of sensitized dorsal horn nociceptive neurons is known to alter the activity of the alpha motor neuron pool, thus creating one type of the muscle guarding reflex (51, 52). For example, stimulation of the kidney, ureter, or colon in rabbits induces variable, paravertebral muscle responses depending on the organ stimulated (53). Additionally, biochemical stimulation of nociceptors via bradykinin, and serotonin, can activate the gamma motor neuron system (54), which excites segmental stretch reflexes, limits muscle flexibility, and can contribute to formation/perpetuation of myofascial trigger points (MTrPs) (55). MTrPs, the hallmark of MPS, are defined as hyperirritable nodules in a taut band of skeletal muscle. They are the principal cause of musculoskeletal pain and are characterized as either active or latent (56). Active MTrPs are known to produce spontaneous local and or referred pain at rest, whereas latent MTrPs do not. Latent MTrPs are typically considered the “dormant,” subthreshold state, of the active MTrP (57). Tender points (TPs) on the other hand, are described as areas of tenderness occurring in muscle, the muscle-tendon junction, bursa, or fat pad, and are typically considered characteristic of fibromyalgia syndrome (58). Although it is to be emphasized that MTrP and TP are separate entities, recent research utilizing intramuscular electromyographic registration of spontaneous electrical activity, has demonstrated that most fibromyalgia TP sites are located *inside* the local and or neurological referred pain patterns of active MTrPs (59) and almost all fibromyalgia TP sites, as specified by the American College of Rheumatology criteria are known MTrP locations (60). This clinically observed overlap in referred pain patterns suggests some degree of shared pathophysiology.

It is important to note that tissue texture abnormalities including MTrPs are not always confined to a single segmental level as activated nociceptors can also expand receptive fields to non-contiguous areas, contributing to the development of MTrPs in distant locations (4). Neurogenic inflammation, muscle guarding reflexes and the potential impact of MTrPs on central sensitization are important concepts in the pathogenesis of MPS according to existing pain hypotheses.

## Recent Myofascial Pain Hypotheses

### The Integrated Hypothesis

Included among the three most prevailing MPS models, is an expansion of Simons' Integrated Hypothesis described by Gerwin et al. in 2004. It proposes that MTrPs are initiated by local acute or chronic myotendinous injuries including unaccustomed eccentric exercise and or sustained work-related strain (61). Sustained muscle contraction, if persistent, theoretically leads to hypoxia “possibly by the development of high pressures within the contracting muscles” which may explain the significant elevation of vasoactive, inflammatory, and algogenic substances demonstrated in active MTrPs (62). Lowered tissue pH, which inhibits the activity of acetylcholinesterase, combined with the release of calcitonin gene-related peptide (causing increased acetylcholine release), would theoretically contribute to the observed increase in motor end plate activity and focal hypertonicity associated with MTrPs (63). This cycle, if combined with “other factors that predispose to focal hypoperfusion” including “sympathetic nervous system involvement” could be self-sustaining, and unless interrupted, could lead to the initiation and perpetuation of active MTrPs. For multiple reasons, including those discussed in the next section, the integrated hypothesis is no longer considered well-supported by the existing literature.

### The Neurogenic Hypothesis

Theoretical and clinical limitations related to the integrated hypothesis led to the development of the Neurogenic Hypothesis in 2010 (63). Srbely also published a second paper (62) contrasting the Neurogenic Hypothesis with the Integrated Hypothesis as it relates to MPS. Srbely noted that MTrPs are linked to non-musculoskeletal conditions and exist in the absence of precipitating mechanical injury. This includes urogenital syndromes (64), and a documented case of herpes zoster infection in which the MTrP resolved following antibiotic therapy (65). Additionally, infectious, psychogenic, and endocrine causes have been attributed to MTrP formation (66), which cannot be adequately explained by the persistent release of acetylcholine or increased motor endplate activity that follows mechanical injury. He hypothesized that MTrPs are neurogenic expressions of central sensitization, potentially evoked and maintained by an underlying primary pathology (e.g., osteoarthritis or visceral disease) located within the common neurologic segment of the MTrP. The local, anatomic, and physiologic changes observed at MTrP sites, he argued, are due to neurogenic inflammation, triggered by segmentally linked, central sensitization. Srbely states that these neurogenic and inflammatory mechanisms could also account for some of the biochemical changes documented in MTrPs during interstitial sampling studies, including decreased pH and increased concentrations of SP (67). The autonomic effects related to MTrPs, he postulated, may also be attributed to central sensitization (68). Subsequently, the Neurogenic Hypothesis expanded the potential causes of MPS to include pathological non-muscular tissues (e.g., degenerative joints) and visceral structures owing to the anatomic convergence of sensory pathways in the dorsal horn. The importance of chronic primary pathologies in driving this pathophysiologic process highlights the need to understand the mechanisms and origins of potential nociceptive sources that contribute to the maintenance of an *ongoing state* of central sensitization. Although the Neurogenic Hypothesis integrates well-established physiologic mechanisms (central sensitization and neurogenic inflammation) to characterize the pathophysiology of chronic inflammatory muscle disease, it currently lacks sufficient supporting evidence in human models.

### The Neuro-Fasciagenic Model of Somatic Dysfunction

Fascia has also been implicated in the formation and maintenance of chronic pain states. For example, Tozzi, in 2014 published an article describing the structural, functional, and neurological properties of fascia arguing that a purely fascial-based rationale could be developed to explain the palpable features (tissue texture changes, asymmetry, restriction of motion, and tenderness) associated with somatic dysfunction (69). He reviewed the existing literature describing over 50 fascial-based factors including neuromuscular, structural, mechanical, fluid, electromagnetic and hormonal influences that may combine “through various types of interactions” to create somatic dysfunction. This manuscript highlighted the considerable body of research which suggests that fascia may be involved in the development of somatic dysfunction and chronic pain states. However, the *specific mechanism* by which fascia contributes to the creation of an ongoing nociceptive source, was not described by Tozzi.

## Interstitial Inflammatory Stasis Hypothesis

### Somatosympathetic Reflexes

For our IIS hypothesis it is important to recognize the role the sympathetic nervous system (SNS) plays in the generation and maintenance of chronic pain states and idiopathic visceral/vascular dysfunction. Stimulation of A delta and C small fiber, unmyelinated nociceptors from virtually all tissue types, reach lamina I and deep into the dorsal horn where they can produce varying degrees of pre and post-ganglionic sympathetic responses termed *somato-sympathetic and/or visceral-sympathetic reflexes* (70). Activation of these reflexes results in the release of NE from postganglionic neurons, which generally elicits peripheral vasoconstriction responses (23), which have been shown to reduce local muscle blood flow (perfusion) by up to 25% (24). These reflexes are the neurological link between peripheral nociceptors and the SNS, and involve segmental, medullary, and/or supramedullary structures (70–72). Experimental evidence supporting SNS involvement in chronic pain states includes reduced muscle perfusion demonstrated in chronic myalgia patients (14), and decreased (improved) spontaneous electromyographic activity recorded from MTrPs, after local injection of a sympathetic antagonist (15). Additionally, the electrical activity in a MTrP locus was shown to increase after emotional stress and was also successfully abolished following local, alpha-adrenergic blockade (16).

It is known that muscle tissue receives both vasoconstrictive and vasodilatory innervation; however, neurogenic vasodilation *has not* been demonstrated in *resting human muscle* tissue [for review see (7, 73)]. Therefore, following nociceptor stimulation and NE exposure (from somato/visceral-sympathetic reflex activity), peripheral vasoconstriction will override the effects of any local vasodilatory neuropeptides (e.g., from neurogenic inflammation). The resultant sympathetic nerve activation (SNA) may lead to disturbances in arterial and venous microcirculation which have been documented in myalgia patients (74, 75) with observed morphological changes including swollen endothelial cells and the local destruction of myofilaments (76). These microcirculatory disturbances have also been identified in MPS patients, within the MTrP locus (77) and are specifically characterized by local muscle hypoxia and reduced washout of inflammatory substances (7). Additional confirmation of vasoconstriction at active MTrP sites was documented utilizing diagnostic ultrasound to analyze the vascular environment surrounding MTrPs. The study concluded that active MTrPs have a constricted vascular bed including an enlarged overall vascular volume indicating venous stasis (20). In this state of impaired venous return, peripheral pro-inflammatory cytokine concentrations can reach the threshold necessary to further stimulate local, chemosensitive nociceptors, establishing a vicious feed-forward cycle which facilitates MTrP formation via nociceptor/DRG sensitization, and continued activation of somato-sympathetic reflexes. The mechanism described is a reflexive, segmental phenomenon that does not require *supraspinal* sympathetic activation and can act as a primary factor in the development of IIS.

In addition to local SNA from somato/visceral-sympathetic reflexes, psychological considerations are also critical to the development of an accurate and comprehensive pain model (Figure 2) as chronic stress may lead to increased peripheral inflammation and NE production. Transient activation of the hypothalamus-pituitary-adrenal axis (HPA) is the body's normal response to an acutely stressful or traumatic event. However, a *chronic* trauma/stress related disorder like post-traumatic stress disorder is associated with dysregulation of the HPA with resultant elevations of plasma NE (78), cerebral spinal fluid IL-6 levels (79), and cerebral spinal fluid substance P levels (80). The additional long term physiological effects of chronic stress include decreased cortisol production with a subsequent elevation of plasma IL-6 levels (81, 82), which may increase the risk of developing cardiovascular disease and or autoimmune disorders (83–85). Elevated levels of IL-6, IL-17A and a dysregulated HPA have also been observed in fibromyalgia patients demonstrating additional overlap between chronic pain states and idiopathic organ/endocrine dysfunction (11, 86). Taken together, these findings highlight the potential contribution of psychological factors in the development of systemic inflammation. In the context of this manuscript, the known correlation between chronic pain and post-traumatic stress disorder may be of significance as elevated levels of IL-6 (inflammation) and NE (vasoconstriction) would create the ideal interstitial environment for the development of IIS.

### Sampling Studies of Chronic Myofascial Pain Patients

In direct support of the IIS concept, sampling studies of interstitial fluid have identified catecholamines and various algesic substances in MPS patients. Microdialysis sampling of interstitial fluid in the locus of active MTrPs has demonstrated lower pH levels and elevated levels of inflammatory mediators including bradykinin, substance P, TNF-a, IL-1b, IL-6, interleukin-8, serotonin, and NE when compared to latent MTrPs and/or controls (12). Elevated NE levels are direct evidence of SNA and local vasoconstriction in active MTrPs. With regards to fibromyalgia, a review of 25 selected studies revealed higher serum levels of IL-6 vs. controls (11). Neuropeptides have also been identified in cerebrospinal fluid (CSF) in response to noxious stimuli. For example, elevated levels of substance P were observed within the CSF of fibromyalgia patients (87) at levels up to three-times greater than healthy controls (88). Additionally, fibromyalgia patients have been found to have significantly increased CSF concentrations of NGF (89), interleukin-8 (90) and intrathecal glutamate (91). Sensitization of the dorsal horn due to nociceptor activation following IIS can result in glial cell activation, and subsequent release of pro-inflammatory mediators in the CSF, mediating the transition from acute local pain to chronic widespread pain. For example, the potential contribution of IIS to CSF inflammation specifically offers a pathophysiological rationale for post-traumatic fibromyalgia syndrome (92). We emphasize that the theoretical contribution of IIS to CSF inflammation in fibromyalgia patients does not preclude additional sources of CNS inflammation in fibromyalgia including neuroendocrine contributions. The potential relationship between IIS and elevated levels of NGF in fibromyalgia patients will also be covered in the *subthreshold endplate potential* section to follow.

### Newly Identified Interstitial Pre-Lymphatic Pathways

In 2018 (93), researchers utilizing confocal laser endomicroscopy, identified previously undescribed interstitial, *pre-lymphatic* sinuses or pathways in the dermis, vascular adventitia, submucosa of the viscera, bronchi, adipose tissue and in *all* fascial tissues of the musculoskeletal system. These macroscopically visible, fluid-filled spaces were confined by thick, well-organized, collagen bundles and have no previously described anatomical correlate. It was further described as a “compressible and distensible” interstitial space in which interstitial fluid or *pre-lymphatic fluid* accumulates and flows. Interestingly, the pathways were primarily associated with tissues involved in frequent movement such as the musculoskeletal system, lungs, and/or digestive tract. The peristaltic nature of these tissues would ostensibly augment the normal movement of interstitial flow created by the circulatory system. Notably, the authors stated that these pre-lymphatic pathways would have important implications in tissue function and pathology including edema, metastasis, disease, and fibrosis. They cited specific examples of impaired interstitial flow, the pathophysiology of which could be explained by occlusion of these pre-lymphatic channels including, characteristic duct edema present in acute bile duct obstruction, and the enlarged extracellular spaces noted in keloid scarring (94). With regards to our hypothesis, if lymphatic pathways are impeded by scarring or tissue contraction (discussed in sections to follow), areas *inflammatory stasis*, may be created capable of continuously stimulating chemosensitive nociceptors (e.g., visceral, vascular, musculoskeletal), and thus act as an ongoing nociceptive source to the CNS.

### The Lymphatic Pump (Intrinsic) Mechanism and Interstitial Inflammatory Stasis

In the lymphatic system, pre-lymphatic channels connect to initial lymphatics vessels which are composed of a thin layer of endothelial cells, although completely lack muscle cells (95). They are physically tethered to the surrounding tissue structure through anchoring filaments (96) thus can be impacted by tensions in the surrounding extracellular compartment. More proximally, lymph fluid empties into collecting lymphatic vessels which contain smooth muscle cells and contain unidirectional valves to prevent retrograde flow. The primary mechanism of lymphatic propulsion is provided by *lymphangions* which are the specialized, contractile segments of lymphatic collecting vessels. Therefore, lymphatic fluid is independently and actively driven by rhythmic, phasic, heart-like contractions of successive lymphangions (defined as the muscle segment between successive valves) eventually emptying into the venous circulation (97). Lymphatic vessels are critically modulated by fluid pressure and inflammatory mediators. As such, lymphatic vessels act to resolve the *inflammatory process* by increasing lymphangion contractile frequency in response to inflammation (98). This lymphatic, homeostatic, clearing mechanism, has been demonstrated in response to multiple inflammatory mediators including substance P, CGRP, neuropeptide Y, vasoactive intestinal polypeptide, prostaglandins, IL-1b and TNF-a (99–101).

Despite the action of this intrinsic anti-inflammatory mechanism, pathophysiological disruption of normal lymphatic propulsion is known to occur, leading to excess inflammation in the extracellular compartment. For example, lymphatic dysfunction has been identified in human patients suffering from inflammatory bowel disease (e.g., Crohn's and ulcerative colitis) as evidenced by lymphatic vessel obstruction, dilation, and submucosal edema (21). Notably, surgical resection of diseased areas, returns the morphological appearance of lymphatic vessels to normal supporting the concept of a *functional* lymphatic disturbance (102). Recent research has shed light on this phenomenon as it is now known that specific cytokines, namely IL-1b, IL-6 and TNF-a, can actually *disable* the normal lymphatic pump mechanism during acute inflammatory events, creating a “dramatic, rapid reduction in lymphatic propulsive flow and frequency (22).” This may occur to prevent the spread of infectious and/or inflammatory agents beyond the region needed for a localized immune response; however, it results in lymphatic stasis. The importance of this research to our IIS hypothesis will become apparent in the following sections as these specific cytokines, trapped in the interstitium, may facilitate the transition from acute to chronic pain by long-term impairment of the local lymphatic pump mechanism.

### Fascial Contractility

The Foundation of Osteopathic Research and Clinical Endorsement or FORCE group has recently written several articles intended to develop a modern definition of fascia. “The fascia is any tissue that contains features capable of responding to mechanical stimuli. The fascial continuum is the result of the evolution of the perfect synergy among different tissues, liquids, and solids, capable of supporting, dividing, penetrating, feeding, and connecting all the districts of the body: epidermis, dermis, fat, blood, lymph, blood and lymphatic vessels, the tissue covering the nervous filaments (endoneurium, perineurium, epineurium), voluntary striated muscle fibers and the tissue covering and permeating it (epimysium, perimysium, endomysium), ligaments, tendons, aponeurosis, cartilage, bones, meninges, involuntary striated musculature and involuntary smooth muscle (all viscera derived from the mesoderm)” (103). Fascia is composed of cells including macrophages and mast cells (104); however, its foundational cell is the fibroblast which is the principal cell responsible for production of the ECM. As cited previously, virtually all fascial tissues (viscera, ligaments, nerves, disc tissue etc.) contain unmyelinated nociceptors, and thus have the potential to become primary nociceptive sources. Cytokines, including the IL-6, TGF-β1, and IL-1β have a significant impact on fibroblasts, stimulating them to differentiate into *myofibroblasts*, a contractile form expressing α-smooth muscle actin. These contractile cells are associated with pathological conditions including palmar fibromatosis and hypertrophic scarring (105). Importantly, myofibroblasts have also been identified in normal, *non-pathological* tissues including the fascia cruris (106), ligaments (107), tendons (108), bronchial connective tissues (109), organ capsules (110), and several other collagenous connective tissues (111). Following inflammatory exposure, myofibroblasts are known to secrete additional cytokines including TGF-β1, IL-1β, etc. which may increase the rate of ECM synthesis, creating fibrosis (112). The production of the cytokine TGF-β1 by fibroblasts takes on additional clinical significance as *normal* (non-pathological) fascia samples were recently demonstrated to contract following TGF-β1 exposure. Both rat and human samples of the lumbar fascia, plantar fascia, and sections of the fascia lata were analyzed and found to contain significant numbers of myofibroblasts. Following application of TGF-β1 to the lumbar fascia, tissue contractions were measured and calculated to be at an estimated force of 2.63N (113). The potential clinical impact of this contraction is below the threshold for mechanical spinal stability; however, is above the threshold for mechanosensory stimulation impacting gamma motor neuron activity and therefore musculoskeletal function (114). Adding additional support to this concept, Schleip found a strong positive correlation between myofibroblast cell density and contractile response, with a generalized increase in myofibroblast density in perimysial tissues (where most spindle capsules are embedded) (113). This corresponds with previous research demonstrating perimysial changes in myofascial pathologies (115) and supports the hypothesis of Stecco et. al. that MPS could be influenced by abnormal perimysial fascial stiffness (116). Important to our hypothesis, the fascia-myofibroblast contractile responses measured following TGF-b1 expression may be capable of partially or fully occluding pre-lymphatic flow through initial lymphatic vessels and/or “compressible” interstitial pathways as described previously by Benias et al. (93). This process may act as an independent, purely *fascial-based mechanism*, capable of disrupting interstitial lymphatic drainage, creating localized regions of IIS.

### Inflammatory Stasis and Fibrosis

Fibrosis or scar tissue formation is defined as thickening of the ECM that is preceded by inflammation or physical tissue injury. Since the same pro-inflammatory cytokines (IL-6, TGF-β1 etc.) involved in our proposed interstitial stasis model are also the exact cytokines described in the process of excessive, non-physiological scar tissue formation (fibrosis), it is plausible there is a shared pathophysiology. Increased ECM synthesis by fibroblasts in response to inflammation is known to cause fibrosis but may also cause the formation of fibrotic clusters called *fibrotic foci* (117) which are associated with idiopathic lung fibrosis. The overproduction of collagen I by myofibroblasts, severely impairs regional tissue architecture and is considered the key component in all types of organ fibrosis (118). In fact, a similar mechanism (to our described hypothesis) has been previously observed in the scarring process citing excessive neuroinflammatory stimuli, prolonged production of growth factor TGF-b1 and overproduction of the ECM (119–121). Since chronic inflammation is the driving force behind myofibroblast proliferation, interruption of the inflammatory process can resolve the fibrotic process (118). For example, viral clearance by interferon, prevents associated liver fibrosis in viral hepatitis patients. Unfortunately, many forms of fibrosis following injury and/or infection are idiopathic (e.g., idiopathic pulmonary fibrosis and/or kidney fibrosis) and intractable as the source of chronic inflammation is unknown. Recognition and resolution of the feed forward, multi-tissue, hypothetical IIS mechanism we describe, by manipulative or pharmacological interventions, may help resolve the symptoms associated with non-physiological scarring and potentially interrupt the process of idiopathic organ fibrosis.

### The Role of Inflammatory Interstitial Stasis in the Generation of Subthreshold Potentials

Subthreshold action potentials (SAPs) generated from areas of IIS may also play a significant role in the process of central sensitization, chronic pain, and the development of *latent* MTrPs. Pro-inflammatory cytokines (including IL-1b and TNF-a), released in response to tissue stressors and/or immune responses, strongly induce nerve growth factor (NGF) synthesis (122). NGF receptor activation and signaling alters nociception via direct nociceptor sensitization at the site of injury and can change gene expression in the DRG, which collectively increases nociceptive signaling from the periphery to the CNS (123). Considering that NGF production is related to peripheral cytokine exposure, NGF production would logically be more likely to occur in zones of IIS. Elevated pro-inflammatory cytokines concentrations would induce NFG production in dorsal horn glial cells, creating SAPs (124). This may have clinical significance as elevated levels of NGF have been identified in the CSF of fibromyalgia patients (89). Therefore, latent MTrPs may be clinical manifestations of *sub threshold* pro-inflammatory cytokine concentrations in areas of IIS. Although latent MTrPs are not associated with spontaneous pain, they can cause local and even referred pain upon deep palpation. Mense hypothesized that latent MTrPs send nociceptive, subthreshold signals toward the dorsal horn of the spinal cord (125), which would effectively cause central sensitization *without* the perception of pain. He emphasized that latent MTrPs may be of particular importance in chronic myalgia as pathological changes in muscle tissue are typically associated with subthreshold input and low frequency activation of nociceptors (125).

To summarize, latent MTrPs may be related to SAPs generated in response to low level pro-inflammatory cytokine exposure in lesser areas of IIS. These nociceptive signals could both initiate and/or maintain central sensitization. The involved tissues and neuromeric fields would logically be prone to injury and or may become symptomatic (suprathreshold) following any additional trauma and/or inflammatory insult.

### Hypothesis Summary (Interstitial Inflammatory Stasis)

Based on the research presented, a novel lymphatic and fascial-based hypothetical mechanism can be described, having major implications in chronic pain states and idiopathic organ syndromes. Tissue injury and/or inflammation from immune responses causes the release of cytokines into neighboring tissues. This inflammatory reaction triggers fibroblasts to release additional cytokines including IL-1b, IL-6 and TGF-β1 thereby exacerbating the local nociceptive and inflammatory processes. These specific cytokines simultaneously disable the local, lymphatic pump mechanism. If interstitial concentrations of IL-1b, IL-6 and or TNF-a reach the threshold necessary to cause significant, local expression of TGFb-1, lymphatic propulsion may become impaired due to fascial (myofibroblast) contraction and or vessel fibrosis. These specific algogenic cytokines, now trapped locally in the interstitium, may continuously stimulate chemosensitive nociceptors and facilitate the transition from acute to chronic pain by long-term impairment of the *local lymphatic pump mechanism*. This is despite eventual recovery of the *systemic lymphatic pump*. The resultant cytokine exposure will also activate local somato/visceral-sympathetic reflexes, impairing regional vascular perfusion. Therefore, this hypothetical, pathophysiological hemodynamic process is due to a combination of impaired vascular perfusion and long-term disruption of the *local lymphatic pump* mechanism. The areas of interstitial stasis generated may exist in any one of the newly identified musculoskeletal, visceral, adventitial and/or dermal interstitial pathways. The resultant stasis and elevated interstitial cytokine concentrations may create a feed-forward nociceptive loop, which results in continuous stimulation of musculoskeletal and or non-musculoskeletal nociceptors, maintaining the process. The hypothesis is not selectively dependent on pathology and or any specific source of inflammation, as multiple tissue nociceptors are capable of initiating and maintaining IIS.

Applying the IIS hypothesis to musculoskeletal pain research, the cytokines (IL-1b, IL-6, TNF-a) shown to disable the lymphatic pump mechanism are the *exact cytokines* found to be primarily involved in the perception of pain (24), fascial contraction following TGF-b1 production (22, 105), and were also among those elevated in active MTrP sampling studies (11, 12). Additional experimental support for the IIS hypothesis is the fact that long-term, impaired lymphatic drainage was recently identified in the pathogenesis of lymphedema. Histological examination of lymphatic vessels in 29 secondary lymphedema patients demonstrated “contracted-type” and “sclerotic-type” collecting vessels in areas of lymphatic stasis. These vessels were found to have occluded lumens causing impairment of the normal lymphatic-pump mechanism. Most significantly, many of the contractile cells responsible for impairing lymphatic drainage were identified as *myofibroblasts*, not vascular smooth muscle cells, and were characterized by increased ECM synthesis (126). Also in support of the IIS hypothesis is a finding of Asano et al. who demonstrated increased levels of inflammatory cytokines, namely TNF-a and IL-1b, in the walls of dysfunctional lymphatic collecting vessels (127). This lead Carthy to suggest that the *transformation into myofibroblasts* may have been triggered by local inflammation (128). Considered collectively, these recent research findings from the field of lymphedema, lend support to the hypothesis that ongoing impairment of the normal lymphatic pump mechanism (IIS) being involved in the generation and maintenance of chronic pain states.

Using irritable bowel syndrome as a *non-musculoskeletal* example, compelling evidence exists that increased inflammation in the enteric mucosa or neural plexuses may initiate the development of IBS-like symptoms (129). In a recent study of acute gastroenteritis infection patients, 23% were found to develop IBS-like symptoms within 3 months after infection. Altered gut physiology including evidence of chronic inflammation was still present at 3 months in both the symptomatic and asymptomatic groups, implicating post-infectious *peripheral inflammation* as a contributing factor. When the symptomatic and asymptomatic patients were compared based on psychosocial factors, elevated stress profiles (potentially, HPA dysregulation) were strongly associated with those who would eventually develop IBS-like symptoms (13). In this example, elevated sympathetic drive from stress would feed into the peripheral stasis mechanism we describe. As the associated elevation in NE and IL-6 levels (related to chronic stress) may induce vasoconstriction, reduce lymphatic propulsion, increase fibroblast to myofibroblast differentiation and create nociception. This emphasizes the fact that both peripheral and central factors must be considered in idiopathic pain states.

Based on the findings presented, we propose the following feed-forward pathophysiological hypothesis for MPS, and certain idiopathic visceral/vascular conditions. Figure 1 is a simplified view of our IIS hypothesis including only biochemical, sympathetic, fascial, and lymphatic influences. Figure 2 is a *comprehensive pain model* detailing multiple factors (including those described in Figure 1) related to the development of IIS and the subsequent pathophysiological outcomes.

## Discussion

Our proposed hypothesis expands existing pain models by highlighting the mechanisms by which IIS may be initiated and act as an ongoing peripheral nociceptive source. Via this mechanism, fascial, visceral, vascular and or neural pre-lymphatic pathways may entrap inflammatory mediators, which would continually stimulate local nociceptors, contributing to central sensitization, chronic pain, and sympathetic activation. Importantly, algesic substances trapped in the interstitium (not blood stream), have the potential to create a state of recalcitrant, *non-healing* pain, that may be resistant to pharmacological intervention.

As Figure 2 demonstrates, the precipitating factors and pathophysiological mechanisms behind each patient's symptoms are unique and may often occur in combination within the same neuromeric field. Therefore, the neurological concepts of temporal and spatial summation would have important implications in the proposed model as pain can be initiated by a single repeated stimulus over time (temporal summation) or by multiple different pain generating mechanisms *converging* onto the dorsal horn (spatial summation). Even in cases of known pathology, patients may be asymptomatic (e.g., the single nociceptive source fails to override the inhibitory pain system) or may *become* asymptomatic following successful treatment of convergent, non-pathological, nociceptive sources. Therefore, assessment and treatment of all potential pain-producing tissues and mechanisms, as suggested by the proposed model, improves the likelihood of a patient reaching the goal of pain free function. Additionally, an argument could also be made for the treatment of *latent* MTrPs which would reduce central sensitization related to SAPs, helping to maintain pain-free function.

As stated previously, the proposed model is not exclusive to peripherally generated chronic pain as IIS may also offer a physiological rationale for idiopathic visceral and vascular dysfunction. Neurovascular bundles from all spinal segments also innervate vertebral and spinal cord vessels, making them capable of inducing *spinal* vasospasm by activating SNA (130, 131). Vasoconstriction of spinal arteries and veins may contribute to the pathophysiology of common disorders including radiculopathies, myopathies, idiopathic neuropathies, degenerative disc disease and/or degenerative joint disease. Theoretically, SAPs (produced by NGF) may also induce segmentally linked vasoconstriction following sympathetic activation which could serve as a possible explanation for the high incidence of spinal degenerative changes in *asymptomatic* individuals (over 73% of subjects tested) (132, 133). Specifically, subthreshold nociceptive signals generated by IIS could create vasoconstriction of segmental arteries that supply the vertebrae, leading to asymptomatic or *silent* spinal degeneration over time.

Persistent nociceptive input from IIS may also directly impact cranial tissues innervated by the spinal trigeminal nucleus which receives afferent input from the upper 3 cervical segments. In support of this concept, nociceptive inputs into the spinal trigeminal nucleus, including those produced by MTrPs, have been implicated in tension-type headache (19) and may logically, via SNA, contribute to other idiopathic cranial disorders including post-concussion syndrome. Additionally, second order nociceptive neurons projecting to higher centers through the dorsal column, can activate the “brain-gut axis” which links the autonomic nervous system to the neuroendocrine, immune, and enteric nervous systems (134). This could interfere with the normal efferent innervation of the viscera causing abnormal hormonal secretion and/or disruption of gastrointestinal motility (17). Collectively, these findings suggest that alleviating ongoing nociceptive sources related to interstitial stasis may be able to resolve the underlying pathophysiological mechanism responsible for idiopathic spinal, digestive, endocrine and cranial disorders.

### Potential Non-pharmacological Interventions (Related to the Proposed Model)

The need for effective non-pharmacological interventions for pain is increasing with efforts to reduce opioid addiction. One promising intervention purported to deactivate nociceptors and alleviate tissue inflammation is *Counterstrain* (previously called Strain and Counterstrain) (135). Counterstrain utilizes cutaneous TPs/MTrPs to diagnose and treat MPS and idiopathic conditions. Once a TP is located, the body is gently placed into specific positions of ease that have been clinically identified to alleviate TP tension and tenderness. Tissue decompression (through positioning or local tissue manipulation) is believed to silence activated nociceptors, reducing the afferent barrage to the dorsal horn. Reduced nociception, deactivates segmental muscle guarding reflexes, reducing myofascial tension and capillary pressure. The treatment position is then maintained for up to 90 s to allow regional inflammation (interstitial pro-inflammatory cytokines) to gradually dissipate. Based on our hypothetical model, the associated reduction in interstitial NE concentrations during the release would also deactivate somato/visceral-sympathetic reflexes, helping to restore arterial and venous perfusion. Simultaneous reductions in IL-1b, IL-6 and TGF-b1 concentrations would normalize lymphatic propulsion and reduce myofibroblast (facial) contraction blocking pre-lymphatic pathways.

The impact of Counterstrain on inflammation has been investigated at the cellular level, demonstrating improvements in tissue morphology. Researchers repetitively strained human fibroblasts for 8 h in a two-dimensional tissue matrix while measuring the effects on fibroblasts, including cytokine production. A 60-second Counterstrain (or indirect osteopathic manipulative treatment) was then applied which produced beneficial effects on fibroblast morphology, reversing the inflammatory effects (46% reduction in fibroblast IL-6 production after 24 h) when compared to control (136). Recently Counterstrain has been renamed *Fascial Counterstrain* and expanded to include over 800 anatomically named structures, treatments, and diagnostic TPs. This pain-free, non-invasive treatment warrants further investigation as it may have the capacity to alleviate microvascular stasis in all tissues, breaking the feed-forward cycle that creates myofascial pain and potentially idiopathic visceral/vascular syndromes.

Acupuncture, unlike Counterstrain, does not directly target peripheral inflammation (IIS) but is purported to work by dampening nociceptive input to the dorsal horn. Melzack and Wall's gate theory (137) proposes that the superficial dorsal horn of the spinal cord can be excited or *opened* by nociceptors and *closed* by stimulation of large A-beta nerve fibers. Since electroacupuncture is known to stimulate A-beta fibers (138) it is presumed that acupuncture works by activating this pain-gating mechanism. Alternatively, manual acupuncture is known to stimulate A-delta fibers (139) that synapse directly with inhibitory interneurons within the dorsal horn and can inhibit central pain transmission through enkephalin-dependent mechanisms (140). Recent studies of a similar intervention, termed dry needling, have demonstrated antinociceptive effects when treatments were targeted segmentally to discrete MTrP locations as compared to non-MTrP sites (141, 142). Dry needling may also be effective in reducing nociception generated by IIS.

Although the underlying mechanisms driving these interventions remain unclear, it is likely that local and segmentally targeted therapies will be of value in the treatment of chronic pain states generated peripherally by IIS.

### Experimental Validation of the Proposed Model

A central tenet to this hypothesis is the development of a functional disturbance in the lymphatic pump mechanism. The current gold standard for quantifying lymphatic flow includes lymphangiography and lymphoscintigraphy, which have been previously employed to investigate disturbances in the lymphatic pump mechanism including blockage of lymphatic flow (143). More recent technologies, including near-infrared fluorescent optical imaging and/or transit-time ultrasound technique, provide *real-time* quantitative measures of lymphatic flow which could also be employed to identify functional lymphatic disturbances in somatic and/or visceral tissues.

Initial cross-sectional studies comparing clinical cohorts to healthy controls may also be useful in highlighting differences in lymphatic propulsion in support of our hypothesized reduction in lymphatic flow in chronic MPS. We would expect to observe decreased lymphatic flow localized within the region of hyperirritable MTrPs, in contrast to normal tissue. The role of fascial contractures in this mechanism may be further studied by examining for evidence of fibroblast activation biopsied from muscle tissue of fibromyalgia patients (specifically in tissues found to have lowered pain-pressure thresholds). This includes excess TGF-β1 expression, elevated levels of inflammatory mediators, increased myofibroblast concentrations and/or evidence of excess ECM secretion.

These human studies could be followed by controlled animal injury studies to investigate the causal relationships between cytokine accumulation and altered lymphatic flow. Previous animal models have been developed to assess the effect of lipopolysaccharide (LPS) induced production of TNF-a, IL-6 and IL-b (144). These could be used to assess lymphatic stasis utilizing near-infrared fluorescent optical imaging. Immunohistochemistry can be employed to detect TGF-b1 expression (145), which would introduce the potential for fascial contraction and/or fibrosis related to the production of the specific cytokines theoretically associated with IIS. Histological analyses of the fascial tissues could be performed to confirm the presence of fibrotic changes and fascial contractions.

## Data Availability Statement

The original contributions presented in the study are included in the article/supplementary material, further inquiries can be directed to the corresponding author/s.

## Author Contributions

BT was responsible for conceptualization, design, writing, and editing of manuscript. JS and MV was responsible for design, writing, and editing. GR was responsible for design and writing. JS was responsible for design, writing, and editing of manuscript. All authors contributed to the article and approved the submitted version.

## Conflict of Interest

The authors declare that the research was conducted in the absence of any commercial or financial relationships that could be construed as a potential conflict of interest.

## Publisher's Note

All claims expressed in this article are solely those of the authors and do not necessarily represent those of their affiliated organizations, or those of the publisher, the editors and the reviewers. Any product that may be evaluated in this article, or claim that may be made by its manufacturer, is not guaranteed or endorsed by the publisher.
